# Biomarker Utility for Peripheral Artery Disease Diagnosis in Real Clinical Practice: A Prospective Study

**DOI:** 10.3390/diagnostics10090723

**Published:** 2020-09-20

**Authors:** Alexandr Ceasovschih, Victorita Sorodoc, Viviana Onofrei (Aursulesei), Dan Tesloianu, Cristina Tuchilus, Ecaterina Anisie, Antoniu Petris, Cristian Statescu, Elisabeta Jaba, Alexandra Stoica, Elena-Daniela Grigorescu, Irina M. Jaba, Laurentiu Sorodoc

**Affiliations:** 1 Department of Internal Medicine, Clinical Emergency Hospital “Sfantul Spiridon”, 700106 Iasi, Romania; alexandr.ceasovschih@yahoo.com (A.C.); alexandra.rotariu.stoica@gmail.com (A.S.); laurentiu.sorodoc@gmail.com (L.S.); 2 Faculty of Medicine, Grigore T. Popa University of Medicine and Pharmacy, 700115 Iasi, Romania; aursuleseiv@yahoo.com (V.O.); ctuchilus@yahoo.com (C.T.); antoniu.petris@yahoo.ro (A.P.); cstatescu@gmail.com (C.S.); elena-daniela-gh-grigorescu@umfiasi.ro (E.-D.G.); 3 Department of Cardiology, Clinical Emergency Hospital “Sfantul Spiridon”, 700106 Iasi, Romania; dan_tesloianu@yahoo.com; 4 Department of Microbiology/Immunology, Clinical Emergency Hospital “Sfantul Spiridon”, 700106 Iasi, Romania; catianisie@yahoo.com; 5 Department of Cardiology, Cardiovascular Diseases Institute “Prof. Dr. George I.M. Georgescu”, 700503 Iași, Romania; 6 Department of Statistics, Alexandru Ioan Cuza University, 700506 Iasi, Romania; elisjaba@gmail.com; 7 Department of Diabetology, Clinical Emergency Hospital “Sfantul Spiridon”, 700106 Iasi, Romania; 8 Independent Researcher, 700115 Iasi, Romania; imjaba@gmail.com

**Keywords:** peripheral artery disease, atherosclerosis, diagnosis, biomarkers

## Abstract

Peripheral arterial disease (PAD) is a common manifestation of generalized atherosclerosis, which affects more than 200 million patients worldwide. Currently, there is no ideal biomarker for PAD risk stratification and diagnosis. The goal of this research was to investigate the levels of inflammation biomarkers and cystatin C and to explore their utility for the diagnosis of PAD. The study included 296 participants, distributed in two groups: 216 patients diagnosed with PAD and 80 patients without PAD as controls. All studied biomarker levels (C-reactive protein, CRP; fibrinogen; erythrocyte sedimentation rate, ESR; neopterin; beta 2-microglobulin, B2-MG; and cystatin C) were significantly higher in the PAD group and indirectly correlated with the ankle–brachial index (ABI). The final logistic regression model included an association of neopterin, fibrinogen, and cystatin C as the most efficient markers for the prediction of PAD diagnosis. When comparing the area under the curve (AUC) for all biomarkers, the value for neopterin was significantly higher than those of all the other analyzed biomarkers. In agreement with previous studies, this research shows that markers such as fibrinogen, CRP, ESR, B2-MG, and cystatin C have significant value for the diagnosis of PAD, and also clearly underlines the accuracy of neopterin as a leading biomarker in PAD prediction.

## 1. Introduction

Peripheral artery disease (PAD) is a common vascular disease involving major structural and functional changes of the lower limbs’ arterial circulation, leading to disability, a marked decline in quality of life, and high mortality with a substantial economic burden [1,2,3,4,5,6,7]. Presently, the prevalence of PAD is steadily growing, accounting for 2–10% of the general population and up to 20% of patients are over 70 years of age. It reflects the ongoing process of life expectancy prolongation, along with suboptimal correction of the main atherosclerosis risk factors (smoking, arterial hypertension, dyslipidemia, and diabetes mellitus) [8,9,10,11,12,13].

Atherosclerosis represents the main pathophysiological substrate of PAD [1]. Atherosclerotic plaque formation is based on a gradual arterial wall accumulation of lipids and inflammatory cells [14,15]. Progressive endothelial dysfunction also significantly contributes to developing atherosclerosis lesions [16]. Several plasma biomarkers of acute or chronic athero-inflammation have been proposed for the diagnosis of PAD, especially various inflammation cytokines, acute phase reactants, markers of endothelial dysfunction, and subsets of mast cell and monocyte-derived macrophages [17,18].

Various studies have identified the “classical” inflammatory biomarkers as vascular inflammation markers and potential candidates for PAD prediction [19]: C-reactive protein (CRP), a pentraxin family member and an inflammatory cytokine [20]; fibrinogen, a glycoprotein complex and a marker of thrombosis cascades [21,22,23]; and the erythrocyte sedimentation rate (ESR), a non-specific marker of inflammation, increasing with high plasma concentrations of acute phase proteins [24,25].

In recent decades, the “novel” biomarkers panel has gained particular interest. Neopterin, a member of the pteridine family, is an indicator of macrophage activation [26,27]. Several investigators have reported a relationship between neopterin and atherosclerosis plaque progression or instability [28]. Beta 2-macroglobulin (B2-MG), a component of the major histocompatibility complex class I molecule, is an acute-phase reactant protein, whose levels were described as elevated in patients with carotid artery intima-media thickening and arterial stiffness. Cystatin C, a non-glycosylated protein affiliated with cystatin protease inhibitors, is used as either a single marker or as part of a multimarker panel strategy for the assessment of cardiovascular and renal interactions [29,30]. Moreover, high cystatin C levels independently correlate with early atherosclerotic plaque development and progression, even though current studies have primarily focused on carotid and coronary involvement [31,32].

As a result, some biomarkers have already found their application in current clinical practice, but several still require more detailed studies to include them in the diagnosing algorithm of PAD [16,33,34,35]. Therefore, the goal of this research was to investigate the circulating levels of inflammation-related biomarkers and cystatin C and to explore their utility for the diagnosis of PAD.

## 2. Materials and Methods

This study evaluated patients admitted between January 2017 and February 2018 to the Second Department of Internal Medicine and the Department of Cardiology of the Emergency Clinical Hospital *Sf. Spiridon* Iasi, Romania. In this prospective observational study, both PAD and control patients were all consecutively enrolled. A diagnosis of PAD was established in patients with (1) an ankle–brachial index (ABI) value of ≤0.90 and (2) intermittent claudication, or (3) lower extremity arterial revascularization. Individuals who had experienced acute inflammatory diseases in the last 30 days, malignancy, autoimmune diseases, immunodeficient conditions, and neuropsychiatric disorders were excluded from this study.

Medical history data were obtained from the patients’ medical hospital records. Diagnoses of cardio-vascular diseases, diabetes mellitus, and dyslipidemia were previously documented or established in current hospitalization. Hypertension was defined as a systolic/diastolic blood pressure ≥140/90 mmHg or the current use of anti-hypertensive drugs. CAD was determined as a positive history of angina pectoris or prior documentation of ischemic heart disease illustrated by exercise test/echocardiography contractility disorders. Chronic heart failure diagnosis was based on major and minor Framingham criteria. Diabetes mellitus was defined as HbA1c ≥6.5%, a fasting blood glucose level ≥126 mg/dL, or current hypoglycemic medication used for diabetes control. Dyslipidemia was defined as total cholesterol >200 mg/dL, low-density lipoprotein cholesterol (LDLc) >130 mg/dL, triglycerides >150 mg/dL, or a history of treatment for dyslipidemia. The body mass index (BMI) was calculated as the ratio of weight (kilograms) divided by the square of height (meters).

The ankle-brachial index (ABI) was assessed in all patients. The blood pressures of the four limbs were measured using a handheld continuous wave Doppler probe (SonoTrax, San Diego, CA, USA). The ABI was calculated using the ratio of the systolic arterial blood pressure on the posterior tibial and dorsalis pedis arteries divided by the systolic arterial pressure on the brachial arteries.

Doppler ultrasonography was performed using a Samsung Ultrasound System H60 LS22EMU1HS (Samsung Medison, Seoul, Korea). The patients were divided according to the number of arteries that presented various stenosis degrees (mild, <50%; moderate, 50–69%; severe, 70–99%; occluded, 100%) [1,36].

Upon patient admission, blood samples were collected to determine the levels of inflammatory biomarkers and cystatin C. In the laboratory, the serum/plasma samples were isolated by centrifugation and then tested, or frozen at −20 °C for up three months, until the samples were analyzed. The neopterin and B2-MG concentrations were measured using the ELISA method (IBL International GmbH, Hamburg, Germany), CRP was analyzed using the immune turbidimetric assay on the ARCHITECT c16000 automate analyzer (Abbott Laboratories, Wiesbaden, Germany), fibrinogen was evaluated using a fully automated coagulation analyzer (ACL TOP 750) (Instrumentation Laboratory, Bedford, Massachusetts, United States), and ESR was determined by standard routine laboratory procedures using the SRS100/II analyzer (Greiner Bio-One, Kremsmünster, Austria). The cystatin C level was detected by spectrophotometry in the ARCHITECT c4000 automated chemistry analyzer (Abbott Laboratories, Wiesbaden, Germany). Normal and abnormal controls of known concentrations were included in each assay performed. For cystatin C level determinations, grossly lipemic samples and samples with very high triglyceride concentrations were diluted as one part sample and four parts isotonic saline to decrease nonspecific light scattering.

The following cut-off values were used as normal interpretation levels: Neopterin (<10 nmol/L), B2-MG (in men, the reference range was 1.15–3.85 mg/L and in women, it was 0.73–3.56 mg/L), fibrinogen (<400 mg/dL), CRP (<0.5 mg/dL), ESR (<10 mm/1 h), and cystatin C (in men, the reference range was 0.41–0.99 mg/L and in women, it was 0.4–0.99 mg/L).

### 2.1. Statistical Analysis

Continuous variables were tested for a normal distribution using the Kolmogorov–Smirnov test and were presented as the median with an interquartile range (IQR). Categorical variables were presented as frequencies and percentages. Differences between the control and PAD groups were compared using parametric (independent sample t test) or non-parametric (Mann–Whitney U) tests, as appropriate. Spearman’s test was used to investigate the correlations between variables. In certain cases, the logarithmic transformation of data was performed.

The analysis of covariance (ANCOVA) was applied in the attempt to clarify if the decrease of ABI in the PAD group was directly a result of the increase in cystatin C, or more likely due to the inflammation/progression of PAD (and therefore leading to higher levels of cystatin C). ANCOVA was used to control for the effects that continuous variables (biomarkers) such as cystatin C may have over the output of ABI between PAD and non-PAD groups.

Receiver operating characteristic (ROC) analysis was used to investigate the diagnostic performance for biomarker levels in PAD, and the areas under the curve (AUCs) were compared [36].

To assess the performance of candidate biomarkers as prediction factors for PAD, a binary logistic regression was executed for each variable that demonstrated significant differences between the two groups. The significant variables in the univariate analysis were included in the final multivariable logistic regression model.

The method used to estimate the coefficients of the logistic regression was maximum likelihood estimation (MLE) and a Wald test was used to evaluate the statistical significance of each coefficient. Odds ratios (OR) with confidence intervals (CI) were calculated [37]. Internal validation of the final model was performed using the bootstrap resampling method (1000 bootstrap samples). For validation purposes, the model calibration was assessed by Hosmer–Lemeshow goodness-of-fit χ2 statistics and the model discrimination by c-statistics.

Data analysis was performed using IBM SPSS Statistics for Windows (version 19) and MedCalc Statistical Software version 19.4.0 (MedCalc Software Ltd., Ostend, Belgium). All tests were two-tailed and a *p*-value < 0.05 was considered statistically significant.

### 2.2. Ethics Statement

The Research Ethics Committee of the Grigore T. Popa University of Medicine and Pharmacy and the Ethics Committee of the Emergency Clinical Hospital Sf. Spiridon board approved this study, and it was conducted according to the ethical guidelines of the 1975 Declaration of Helsinki Principles. All patients gave written informed consent to participate in this study.

## 3. Results

The study included 296 participants, distributed in two groups: 216 patients diagnosed with PAD and 80 patients without PAD as controls.

Baseline characteristics for study participants, in total and by group as well as comparisons between study participants positively diagnosed with PAD and those without PAD are presented in Table 1. In both groups, patients were predominantly male, older, and current or ex-smokers to a high degree, and had a similarly high prevalence of diabetes, hypertension, coronary artery disease (CAD), dyslipidemia, and chronic heart failure. There was no significant difference in the body mass index (BMI) values between PAD and the control groups. Claudication distance (CD) ABI values were significantly lower for the PAD group.

The biomarker profiles in total and by group as well as comparisons between groups are displayed in Table 2. When compared against the control group, the PAD group showed a significant increase in median values for all inflammatory markers as well as cystatin C (*p* < 0.05).

**Figure 1:**
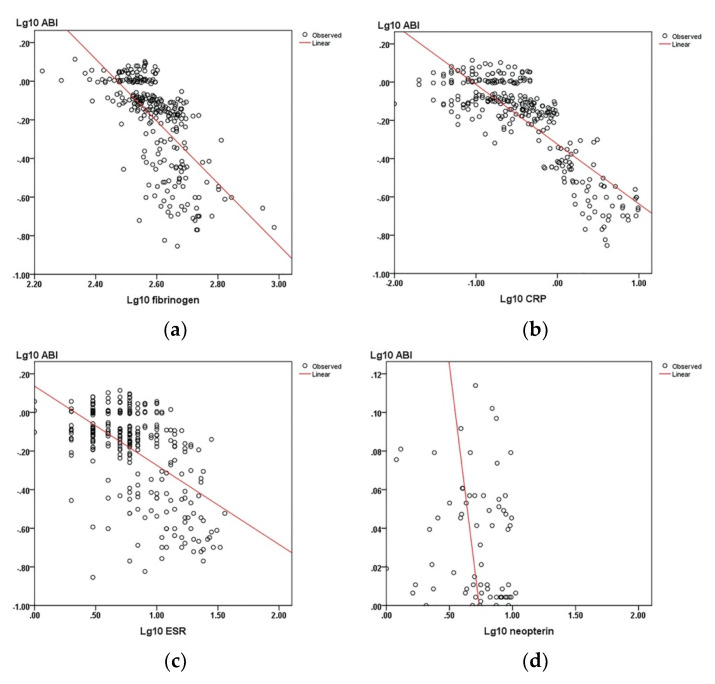

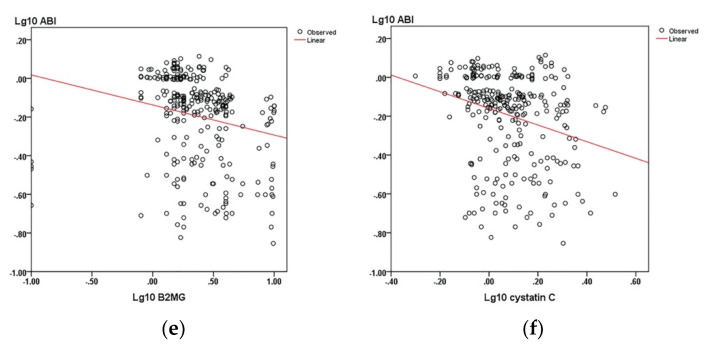
The correlations between the ABI and biomarkers: (**a**) Fibrinogen; (**b**) CRP; (**c**) ESR; (**d**) neopterin; (**e**) beta-2-microglobulin; and (**f**) cystatin C.

As the levels for all biomarkers considered were significantly higher in the PAD group, the next step was to quantify the correlations between these biomarkers and various relevant clinical and sonographic measures of PAD (CD, ABI, and number of arteries with significant stenosis). All correlations between biomarkers and ABI (Figure 1) or the CD were indirect and highly significant (Table 3). All correlations between biomarkers and the number of arteries with stenosis both >50% and >70% were direct and highly significant (Table 3).

All biomarkers had significant positive and direct correlations with each other. The correlation coefficient values were especially high for the association between neopterin and the five remaining biomarkers (Table 3).

ANCOVA results indicated that after controlling for the differences in cystatin C between PAD and non-PAD patients, the differences in ABI (F = 328.179 (1291), *p* = 0.000) between groups remained significant (partial eta square 0.53). The adjusted ABI means and variability (standard error) by group, once controlling for differences in cystatin C as a covariate, were as follows: PAD 0.589 ± 0.13, and non-PAD 1.055 ± 0.22.

### Diagnostic Performance of Biomarkers in PAD

**Figure 2:**
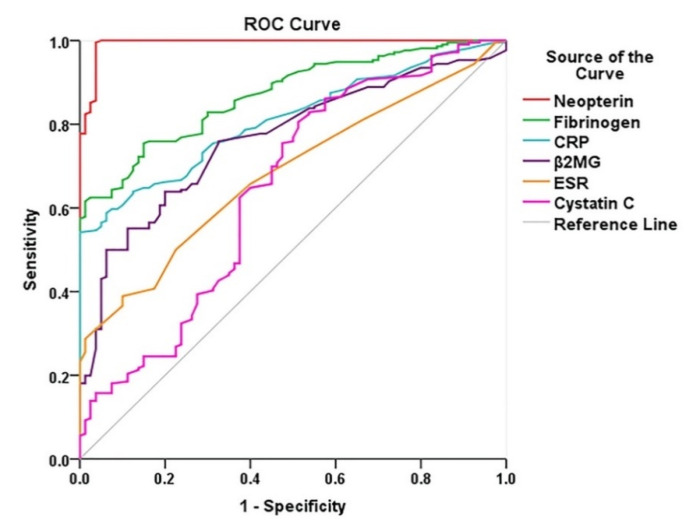
Receiver operating characteristic (ROC) curves for inflammation biomarkers and cystatin C.

The ROC curves show an accurate diagnostic performance for all six biomarkers. The AUCs for neopterin, fibrinogen, and CRP were higher than 0.8 (Figure 2) and the rest of the AUC values were higher or close to 0.7. AUCs for all six biomarkers were highly statistically significant (*p* < 0.05) (Table 4).

AUC values for neopterin were significantly higher when compared with each of the other markers (*p* < 0.0001) (Figure 2).

AUC for fibrinogen is significantly higher than AUC values for cystatin C (*p* < 0.0001), B2-MG (*p* = 0.0024), CRP (*p* = 0.033), and ESR (*p* < 0.0001) (Figure 2).

AUC for CRP was significantly higher than AUC values for cystatin C (*p* = 0.0002) and ESR (*p* = 0.0002). AUC for B2-MG was significantly higher than AUC values for cystatin C (*p* = 0.01) and ESR (*p* = 0.04) (Figure 2).

The results after running a univariate binary logistic regression for each of the biomarkers are presented in Table 5. In these univariate models, all six biomarkers were proven to exhibit significant potential as predictors for PAD risk diagnosis (*p* < 0.05). All markers significant in the univariate analysis were tested in a multivariate logistic model.

Although all variables were predictive for morbidity in univariate analysis, ultimately, some were excluded from the final multivariable logistic regression model, because they did not prove to be statistically significant, namely, B2-MG (mg/L), ESR (mm/1 h) and CRP (mg/dL).

The multivariable analysis indicates that the PAD probability can be best predicted by using the following three biomarkers: cystatin C (mg/L), neopterin (nmol/L), and fibrinogen (mg/dL) (Table 6).

The bootstrap method validates the model, where *p* < 0.001 for all of the prediction variables. The C-statistic was 0.998 (95% confidence interval 0.993-1, *p* < 0.001), validating the discriminatory power of the final model.

## 4. Discussion

One of the main features of peripheral artery disease is the tendency to present constant atherosclerosis progression. The fundamental role of inflammation in the development of atherosclerosis is well-established and many previous studies have thoroughly researched the subject. Nevertheless, there is currently no ideal specific biomarker for PAD, neither for the purpose of risk stratification, nor for a positive diagnosis of PAD. Therefore, this study focused on the relationship between PAD and inflammatory biomarkers as well as PAD and cystatin C in an attempt to reveal a practical hierarchy in their use.

This study represents an accurate model of the daily clinical practice as its control group includes patients without PAD, but with various cardio-vascular disorders. PAD is often associated with other comorbidities such as hypertension, diabetes, and dyslipidemia, and it is important for a biomarker to be related to PAD, even in such a constellation of diseases that are also connected with atherosclerosis, inflammation, and its biomarkers.

In recent decades, many studies in the field of atherosclerosis have used “classical” biomarkers of inflammation separately or as a reference to test the “novel” biomarkers panel. CRP, fibrinogen, and ESR have an especially important significance for PAD diagnosis and risk stratification.

Fibrinogen is an inflammation biomarker of interest, an acute phase reactant, and a determinant of blood viscosity, which has received much attention in the context of PAD [21,22]. The Edinburgh Artery Study researched the whole series of pro-inflammatory biomarkers and revealed that PAD presence and increased levels of fibrinogen had a significant association [38]. In addition, Smith F.B. et al. showed that plasma fibrinogen may promote the development of PAD in the general population [39]. An interesting fact was noted by Unlu Y. et al., who revealed that elevated fibrinogen levels can appear in patients with a high risk of developing symptoms of atherosclerotic disease including PAD [40]. The results of this research are in line with these studies, with fibrinogen levels in the PAD group being significantly higher than those measured in the control group.

CRP has an important role in several aspects of the inflammatory process and quickly responds to its appearance [41]. This CRP property allows it, with a high sensitivity, to detect the tissue damage caused by atherosclerosis in the initial stages [42]. In agreement with results from the National Health and Nutrition Examination Survey [43], this study revealed significantly higher CRP levels in PAD patients compared to the control group.

ESR is an indirect marker of the acute phase reaction [24,44,45]. In this study, ESR values were significantly higher in PAD patients than in the controls. ESR was highly and directly correlated with all of the other inflammation markers, with this association being especially strong for fibrinogen (Table 3). Previous studies have also observed that high ESR values are associated with increased fibrinogen levels [44]. A characteristic of ESR is its relatively slow value change, depending on clinical stage modifications, which explains its rare use as a marker of inflammation in PAD. Another relative disadvantage in using ESR as a marker of inflammation in PAD is the fact that ESR can increase with age, which does not happen with plasma CRP and fibrinogen concentrations [44,46].

When analyzing the association between these “classical” inflammation markers, CD and ABI, this research showed a strong indirect correlation between the levels of fibrinogen, CRP, ESR, and CD/ABI, demonstrating that higher values of inflammatory markers are associated with a significant decrease in CD/ABI (Figure 1). Moreover, several authors that have extensively studied CRP also noticed that its level is independently associated with both ABI and the intermittent claudication distance [38,47,48,49].

According to certain research reports, B2-MG is a biomarker that may be associated with vascular inflammation and arterial stiffness. This study found significantly elevated B2-MG plasma levels in PAD patients compared with the control non-PAD group. B2-MG also indirectly and strongly correlated with CD and ABI. These results are in agreement with similar studies, which demonstrated the same relationship between high B2-MG concentrations and the presence of PAD [50,51,52,53]. On the contrary, Real de Asua D. et al. reported no significant correlation between B2-MG levels and ABI in PAD patients with a high vascular risk [54]. Rheeder P. et al. described similar results; however, their study focused on PAD patients with diabetes mellitus as the comorbidity [55]. In this study, both PAD patients and the control group were characterized by uniform demographic conditions and comorbidities, eliminating the influence of possible confounding factors and confirming the importance of B2-MG in PAD diagnosis.

Given its documented advantages in identifying subclinical kidney disease, cystatin C has constantly been used as a marker associated with cardiovascular events [29,30,56,57]. Additionally, several recent studies have noticed the relationship between serum cystatin C levels and the presence of atherosclerotic plaques [31,32,58]. The proposed mechanism involves an extensive proteolytic activity of the arterial wall extracellular matrix and the compensatory, enhanced activity of cystatin C as a protease enzyme inhibitor [59]. However, there are conflictual reports on the relationship between cystatin C levels and the presumed associated atherosclerotic burden. One valid theory suggests a very dynamic physiology of cystatin C concentrations, dependent upon the stable/unstable pattern of the atheroma plaque [59]. Additionally of interest is the observation by Joosten M.M. et al. that PAD’s association with high cystatin C levels was only noted in the male gender subgroup, or the finding by Liu F. et al., who identified the same relationship in the diabetic population [57,60].

In this context, when investigating cystatin C, significantly increased levels were found for the PAD patients. More importantly, cystatin C was indirectly correlated with CD and ABI, and an increase in its concentration was associated with significant decrease in both CD and ABI values.

In order to attempt clarification of the relationship between ABI and cystatin C in the presence of vascular damage and inflammation, the study tried to establish if the differences observed in ABI between PAD and non-PAD groups were due to the presence of inflammation associated with PAD and not solely and directly to cystatin C. To this end, an analysis of covariance (ANCOVA) was used to control for the differences in cystatin C levels between PAD and non-PAD patients, and to assess whether PAD patients still had decreased ABI values when compared with non-PAD patients.

Controlling the cystatin C impact over ABI showed that the decrease in ABI between PAD and non-PAD patients was maintained and still significant. Partial eta square was 0.53, showing that the variable group (PAD vs non-PAD) was responsible for 53% in the variability of ABI.

This could constitute an argument for the fact that the increase in cystatin C levels in PAD patients is probably secondary to the presence of inflammation and inflammatory markers, and not the main trigger for the ABI decrease.

It is yet to be proven if a causal relation exists between cystatin C and vascular damage. Since the atherosclerosis development and progression involves the inflammatory mechanisms from the early stages, it becomes highly relevant whether the same inflammatory biomarkers used for the diagnosis and prognosis of PAD can, in fact, trigger and promote the endothelial dysfunction [59,60,61].

Interestingly, the inhibiting capacity of increased circulating cystatin C levels over the regenerative properties of the endothelial cells seems to alter the process of angiogenesis and induce endothelial dysfunction. The possible involvement of cystatin C as a negative regulatory mechanism modulating the vascular development has been proposed even from the late embryonic period. Thus, it bears that intricate patterns generate the atherosclerotic plaque burden [29,60,62].

Neopterin is a pteridine produced by activated macrophages and dendritic cells and is able to induce oxidative stress activity [26,27]. Previously published studies have reported neopterin as an important compound of the atheromatous process pathophysiology and considered it a significant atherosclerotic plaque biomarker [16,27]. In agreement with these observations, in this study, the neopterin plasma levels in PAD patients were significantly higher when compared to the control group. The results also revealed an indirect correlation between neopterin levels, CD and ABI, where an increase in neopterin values was associated with a significant decrease of ABI. Similar results have been reported by Signorelli et al. and Barani J. et al. [28,61,62,63,64].

All six biomarkers studied were shown to be indirectly correlated with CD and ABI. Neopterin had the highest value for the correlation coefficient (Spearman rho = 0.9, *p* < 0.01), and as such, the strongest association with the variation of both the CD and ABI. The observed inverse correlation between the biomarker levels and both the CD and ABI values indicate that in PAD, there is a strong association between tissue ischemia (reflected by lower CD and ABI values) and high levels of inflammation markers (both “novel” and “classical”) or cystatin C.

From the imagistic point of view, the number of arteries presenting significant degrees of stenosis was directly correlated with all biomarker levels. The increased levels of both inflammatory biomarkers and cystatin C were associated with higher numbers of stenotic arteries detected, and neopterin and CRP had the strongest association with the arterial stenosis degree (for >70% occlusion, Spearman rho 0.693 and 0.663, respectively; *p* < 0.01), as measured on the Doppler ultrasound (Table 3).

Direct strong correlations were observed between all of the biomarkers involved in the study. These biomarkers cross-influenced each other, and increased levels of one marker were associated with increased concentrations for all of the other biomarkers considered. It was expected that only inflammatory biomarker levels would be directly statistically associated, but interestingly, it was noted that cystatin C also correlated with both “classical” and “novel” inflammatory markers.

When ascertaining the association between these biomarkers, neopterin tended to display the highest values for the correlation coefficient, suggesting that, in the context of PAD, neopterin exerts the strongest direct influence on all of the other markers.

The diagnostic performance for the risk of PAD was compared for all six biomarkers.

ROC analysis was used to investigate the diagnostic performance for biomarker levels. AUC levels for all of the biomarkers studied were very close to or above 0.7, indicating that all are good predictors for PAD. In particular, neopterin and fibrinogen had especially high AUC values (above 0.8), which identified these two markers as the most efficient for discerning PAD diagnosis. When comparing the AUC for all biomarkers, the value for neopterin was found to be significantly higher than all of the other markers analyzed. It became rapidly evident that neopterin had by far the strongest diagnostic performance in pointing to PAD risk among the group of markers considered (Table 4).

Logistic regression was used to predict the diagnosis of PAD based on a group of biomarkers associated more or less directly with inflammation. The final model included an association of neopterin, fibrinogen, and cystatin C as the most efficient markers for the prediction of PAD diagnosis. The odds ratio for neopterin was the highest among all of the markers studied, with a high degree of significance. An increase in neopterin serum levels by one unit increased the risk of PAD by 113 times. Therefore, the logistic regression demonstrates that, among these biomarkers, neopterin was the best important performing predictor for PAD disease (Table 6).

With certainty, an important element in PAD pathophysiology is the endothelial damage [65]. There are various methods for assessing the endothelium dysfunction, but one of the most readily available is the determination of the plasma biomarker concentration [66,67,68]. In this study, the main objective was the investigation of inflammatory biomarkers, which directly reflect the real endothelium status. Based on present research data, the correlations between biomarkers, CD, and ABI values were proven to be indirect and highly significant, confirming that inflammation is one of the major determinant points of endothelial damage in PAD patients.

This study has certain possible limitations. The study population was representative for the northeastern region of Romania and the patients were enrolled from a single tertiary center. Therefore, the population for this study was exclusively Caucasian, and the accuracy when applying these results to populations that include non-Caucasian ethnic groups is limited.

In PAD, biomarkers are tested mainly in establishing risk of progression, to select individuals for diagnostic vascular examination, for prognosis, or in determining the response to therapy.

Unfortunately, biomarkers could not represent a replacement for the physical examination in PAD patient diagnosis and every physician, even in an environment of decreasing time, should perform additional measures (ABI) as part of a physical examination. Further studies are needed in order to identify the most specific biomarker for PAD diagnosis with the same value as, for instance, troponin or brain natriuretic peptide used in other pathology.

In conclusion, over the last two decades, an increasing body of evidence has pointed toward the essential role of biomarkers in evaluating PAD risk. In agreement with previous studies, this research shows that markers such as fibrinogen, CRP, ESR, B2-MG, and cystatin C have significant value for the presence of PAD, and it also clearly underlines the accuracy of neopterin as a leading biomarker in PAD prediction.

## Figures and Tables

**Table 1:** Baseline characteristics of patients with PAD and control participants.

Characteristic	Total (*n* = 296)	PAD (*n* = 216)	No PAD (*n* = 80)	*p*-Value
**Age, years, median (IQR)**	69 (15)	69 (15)	68 (16)	0.709
**Gender, N (%)**				0.773
Women	56 (18.9)	40 (18.5)	16 (20)	
Men	240 (81.1)	176 (81.5)	64 (80)	
**Residence, N (%)**				0.133
Urban	117 (39.5)	91 (42.1)	26 (32.5)	
Rural	179 (60.5)	125 (57.9)	54 (67.5)	
**Cigarette smoking, N (%)**				0.862
Current smoker	73 (24.7)	53 (24.5)	20 (25)	
Ex-smoker	183 (61.8)	135 (62.5)	48 (60)	
Non-smoker	40 (13.5)	28 (13)	12 (15)	
**BMI, kg/m^2^, median (IQR)**	27.14 (6.46)	27.07 (6.25)	27.49 (7.04)	0.628
**Hypertension, N (%)**	274 (92.6)	198 (91.7)	76 (95)	0.332
**Diabetes mellitus, N (%)**	119 (40.2)	89 (41.2)	30 (37.5)	0.564
**Dyslipidemia, N (%)**	278 (93.9)	204 (94.4)	74 (92.5)	0.535
**Coronary artery disease, N (%)**	203 (68.6)	145 (67.1)	58 (72.5)	0.378
**Heart failure, N (%)**	212 (71.6)	152 (70.4)	60 (75)	0.433
**CD, median (IQR)**	200 (18.25–517)	140 (10–240)	800 (600–945)	<0.0001
**ABI, median (IQR)**	0.76 (0.49–0.99)	0.67 (0.36–1.19)	1.025(1.01–1.12)	<0.0001

BMI, body mass index; IQR, interquartile range; N, number.

**Table 2:** Biomarker comparison of PAD and control groups *.

Variables	Total (*n* = 296)	PAD (*n* = 216)	No PAD (*n* = 80)	*p*-Value
CRP, mg/dL	0.35 (0.15–0.9)	0.52 (0.22–1.4)	0.15 (0.08–0.27)	<0.0001
Fibrinogen, mg/dL	386.5 (344–454.75)	417 (367–467)	355.5 (302.25–362)	<0.0001
ESR, mm/1 h	6 (4–10)	6.5 (4–13)	5 (3–6)	<0.0001
Neopterin, nmol/L	11.75 (9.61–21.05)	13.15 (11.17–28.42)	5.58 (3.91–8.11)	<0.0001
B2-MG, mg/L	2.13 (1.6–3.63)	2.62 (1.8–4)	1.6 (1.3–1.94)	0.046
Cystatin C, mg/L	1.16 (0.93–1.48)	1.19 (1.01–1.49)	0.99 (0.86–1.36)	0.001

* Values are expressed as the median (IQR, interquartile range).

**Table 3:** Correlations between CD, ABI, the number of arteries with significant stenosis (>50% and >70%) and biomarkers.

Variables	CRP	Fibrinogen	ESR	Neopterin	B2-MG	Cystatin C
CRP	1.00	0.557 *	0.495 *	0.759 *	0.310 *	0.233 *
Fibrinogen	0.557 *	1.00	0.501 *	0.673 *	0.303 *	0.242 *
ESR	0.495 *	0.501 *	1.00	0.496 *	0.186 *	0.234 *
Neopterin	0.759 *	0.673 *	0.496 *	1.00	0.350 *	0.293 *
B2-MG	0.310 *	0.303 *	0.186 *	0.350 *	1.00	0.192 *
Cystatin C	0.233 *	0.242 *	0.234 *	0.293 *	0.192 *	1.00
CD	−0.765 *	−0.765 *	−0.503 *	−0.900 *	−0.353 *	−0.293 *
ABI	−0.761 *	−0.732 *	−0.505 *	−0.900 *	−0.377 *	−0.302
No of arteries with >50% stenosis	0.528 *	0.508 *	0.319 *	0.524 *	0.299 *	0.354 *
No of arteries with >70% stenosis	0.663 *	0.458 *	0.434 *	0.693 *	0.162 *	0.269 *

* Correlation significant at the 0.01 level (two-tailed).

**Table 4:** Diagnostic test performance for biomarkers in PAD.

Variables	AUC	*p*-Value	95% CI for AUC
Neopterin, nmol/L	0.993	<0.0001	0.985–1.000
Fibrinogen, mg/dL	0.870	<0.0001	0.831–0.910
CRP, mg/dL	0.811	<0.0001	0.763–0.858
B2-MG, mg/L	0.765	<0.0001	0.709–0.821
ESR, mm/1 h	0.682	<0.0001	0.620–0.744
Cystatin C, mg/L	0.645	<0.0002	0.570–0.721

AUC, area under curve; CI, confidence interval.

**Table 5:** Univariate analysis models for PAD risk.

Variables	Coefficient (B)	SE	Exp(B)/Odds Ratio	95% Cl for Exp(B)	*p*-Value
Neopterin, nmol/L	3.771	0.964	43.441	6.563–287.518	<0.0001
Fibrinogen, mg/dL	0.03	0.004	1.03	1.022–1.039	<0.0001
CRP, mg/dL	4.891	0.898	133.135	22.904–773.89	<0.0001
B2-MG, mg/L	0.874	0.166	2.396	1.73–3.317	<0.0001
ESR, mm/1 h	0.186	0.041	1.205	1.112–1.306	<0.0001
Cystatin C, mg/L	1.297	0.382	3.658	1.73–7.734	0.001

CI, confidence interval; SE, standard error.

**Table 6:** Multivariable logistic regression model for PAD risk.

Variables	Coefficient (B)	SE	Exp(B)/Odds Ratio	95% Cl for Exp(B)	*p*-Value
Intercept (Constant)	−62.177	16.764	-	-	<0.0001
Neopterin, nmol/L	4.728	1.331	113.113	8.329–1536.102	<0.0001
Fibrinogen, mg/dL	0.033	0.014	1.034	1.005–1.063	0.019
Cystatin C, mg/L	4.409	2.098	82.155	1.345–5016.741	0.036

CI, confidence interval; SE, standard error.

## Abbreviations

ABI	ankle-brachial index
B2-MG	beta 2-microglobulin
CD	claudication distance
CRP	C-reactive protein
ESR	erythrocyte sedimentation rate
PAD	peripheral artery disease
